# Effect of the coronavirus disease 2019 pandemic on people with Parkinson's disease: experience from a Croatian regional center

**DOI:** 10.3325/cmj.2022.63.62

**Published:** 2022-02

**Authors:** Mario Hero, Gloria Rožmarić, Ena Šukunda, Eliša Papić, Valentino Rački, Vladimira Vuletić

**Affiliations:** 1 Faculty of Medicine, University of Rijeka, Rijeka, Croatia; 2 Institute of Emergency Medicine of the Primorsko-Goranska County, Rijeka, Croatia; 3 Clinic of Neurology, University Hospital Center Rijeka, Rijeka, Croatia

## Abstract

**Aim:**

To assess the effect of social isolation due to the coronavirus disease 2019 (COVID-19) pandemic on physical and mental health of Parkinson’s disease patients treated at the University Hospital Center Rijeka.

**Methods:**

This cross-sectional telephone study involved Parkinson’s disease patients who had at least one control examination at University Hospital Center Rijeka in 2020 and were Croatian citizens. A questionnaire was used to obtain data on the socio-demographic characteristics and the severity of motor, anxiety, depression, and non-motor symptoms.

**Results:**

The final sample included 87 patients. Most patients reported subjective worsening of motor symptoms. Patients who lived alone had worse motor scores than those not living alone. The majority of patients reported worsening of anxiety symptoms. Significant worsening of anxiety symptoms was found in patients who lived alone, had a longer disease duration, and had avoided check-ups. Fewer patients had depression symptoms than motor and anxiety symptoms. Significantly higher Hamilton Depression Rating Scale scores were observed in patients with a longer disease duration. Significant worsening of non-motor symptoms was identified in patients who lived alone, were less educated, had a longer disease duration, and had a higher Charlson comorbidity index.

**Conclusion:**

Patients who live alone, have longer disease duration, are less educated, avoid check-ups, and have more comorbidities are more vulnerable to the negative effects of social isolation.

^4^Department of Neurology, Faculty of Medicine, University of Rijeka, Rijeka, Croatia

The coronavirus disease 2019 (COVID-19) pandemic has thoroughly changed many aspects of human daily life (1). From December 31, 2019 to February 21, 2021, there were 112 348 223 COVID-19 cases worldwide and 36 607 500 in Europe. In Croatia, 240 017 people were infected and 5449 died (1). Poorer outcomes and higher mortality rates have been reported in older adults and people with comorbidities such as cardiovascular disease, diabetes, hypertension, chronic obstructive pulmonary disorder, and chronic kidney disease (2). Recent studies suggest that people with Parkinson's disease have an increased death rate (3,4).

During the pandemic, people belonging to risk groups were forced to limit their social life and reduce the number of contacts. Any change in behavior that greatly alters a person's life requires the flexibility to adapt to new circumstances. This cognitive process depends on dopaminergic function (5). A deficient dopamine-dependent adaptation can lead to feelings of helplessness and increased psychological stress (5). Higher levels of psychological stress can worsen motor and non-motor symptoms of Parkinson's disease, but also trigger psychiatric comorbidities such as depression and anxiety (6). Physical activity can alleviate the progression of Parkinson's disease symptoms, so a lack of activity often worsens motor symptoms (5,7). A further significant problem during the COVID-19 pandemic is avoidance of follow-up examinations (4,5,8,9). Delaying or avoiding medical care may increase the risk of morbidity and mortality associated with otherwise treatable or preventable diseases (9).

The main aim of this study was to assess the effect of social isolation due to the COVID-19 pandemic on physical and mental health in Parkinson’s disease patients treated at the Clinic of Neurology of University Hospital Center Rijeka. The specific aims were to assess the effect of social isolation on 1) non-motor symptoms, 2) the occurrence of depression, 3) the occurrence of anxiety, and 4) avoiding check-ups, in people with Parkinson's disease.

## Patients and methods

### Patients

**Figure 1:**
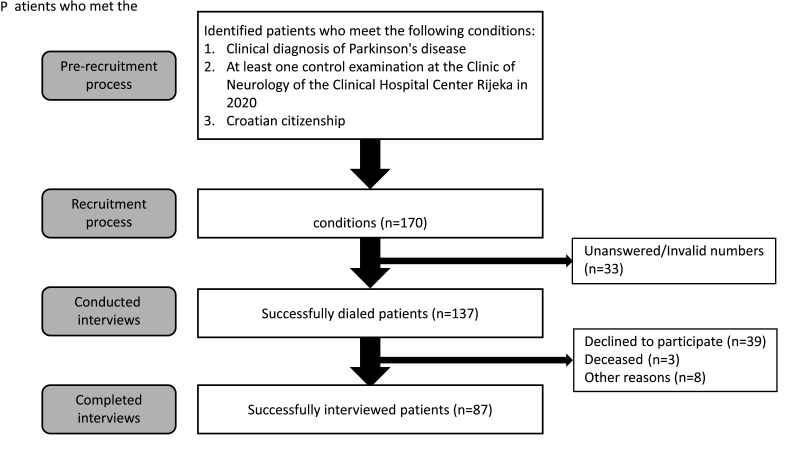
Patient recruitment flowchart.

This cross-sectional study enrolled 170 patients from the outpatient subspecialist clinic. The inclusion criteria were a clinical diagnosis of Parkinson's disease based on the UK Parkinson’s Disease Society Brain Bank Diagnostic Criteria; at least one control examination at the Clinic of Neurology of the University Hospital Center Rijeka in 2020; and Croatian citizenship (Figure 1). The patients' telephone numbers were obtained from the hospital electronic records system, and the patients were attempted to be contacted up to three times on three different days. The telephone questionnaire was conducted from November 15, 2020 to February 15, 2021. The participants gave oral informed consent at the start of the interview, while the written informed consent was obtained later on a control visit or via mail. The study was approved by the Ethics Committee of the University Hospital Center Rijeka.

### The questionnaire

The questionnaire consisted of 75 questions divided into five categories (Supplementary Figure 1(Supplementary Figure 1)). The first category consisted of 10 questions (questions 1-10) on age, sex, duration of illness (in years), education level (primary, secondary, upper secondary, and tertiary education), relationship status, living status (alone or partnered/with family). settlement size (rural regions 0-10,000; semiurban regions 10 000-100,000, large cities >100 000 inhabitants), employment status, presence of comorbidities, COVID-19 positivity (necessary positive test for COVID-19), and avoidance of check-ups (yes/no). The Charlson comorbidity index score is a validated method of classifying comorbidities based on their severity with an aim to estimate short-term and long-term mortality. The second category consisted of four questions (questions 11-14) related to a subjective assessment of the worsening of motor symptoms since the onset of the COVID-19 pandemic. The third category was dedicated to subjective anxiety assessment with the Hamilton Anxiety Rating Scale (HAM-A) (questions 15-28) (10). The fourth category involved a subjective assessment of depression with the Hamilton Depression Rating Scale (HAM-D) (questions 29-45) (11). The fifth category involved the assessment of the symptoms with the modified Non-Motor Symptom Assessment Scale for Parkinson’s Disease (NMSS) (questions 46-75). The patients answered with yes or No. If the patients answered with yes, they were also asked about the worsening of the listed symptoms on a scale of 1-4 (12).

### Statistical analysis

Normality of distribution was assessed with the Shapiro-Wilk and Kolmogorov-Smirnov test. The Mann-Whitney test was used for single comparisons, while the Kruskal-Wallis test was used for multiple comparisons. The Spearman’s rank-order correlation was performed to assess the correlation between four parts of the questionnaire. Statistical significance level was set at *P* ≤ 0.05. The results are presented as medians (interquartile ranges) with corresponding Z- or U-values, where applicable. Statistical analysis was performed with Graphpad Prism 9 (GraphPad Software, San Diego, CA, USA).

## Results

Table 1 summarizes the socio-demographic data. There were 51 women (58.6%) in the final sample. The mean age was 72.9 years, and the mean duration of the disease was 9.6 years. The majority of respondents were married or partnered (71.3%), lived with their spouse or partner (71.3%), and had completed secondary education (90.8%). Patients lived in larger cities (41.4%), rural regions (39.1%), and semiurban areas (19.5%). No patients suffered from COVID-19, and all were retired.

**Table 1:** Socio-demographic data of patients with Parkinson's disease (N = 87)

Variables	No. (%) or mean (standard deviation)
Sex	
female	58.6
male	41.4
Age, years	71.79 ± 8.06
Duration of disease, years	9.506 ± 7.13
Relationship status	
partnered	71.3
alone	28.7
Living status	
partnered/family	71.3
alone	28.7
Level of education	
primary education	9.2
secondary education	41.4
upper secondary education	21.8
tertiary education	27.6
Settlement size	
rural (0-10 000 inhabitants)	39.1
semiurban (10 000-100 000 inhabitants)	19.5
urban (>100 000 inhabitants)	41.4

**Figure 2:**
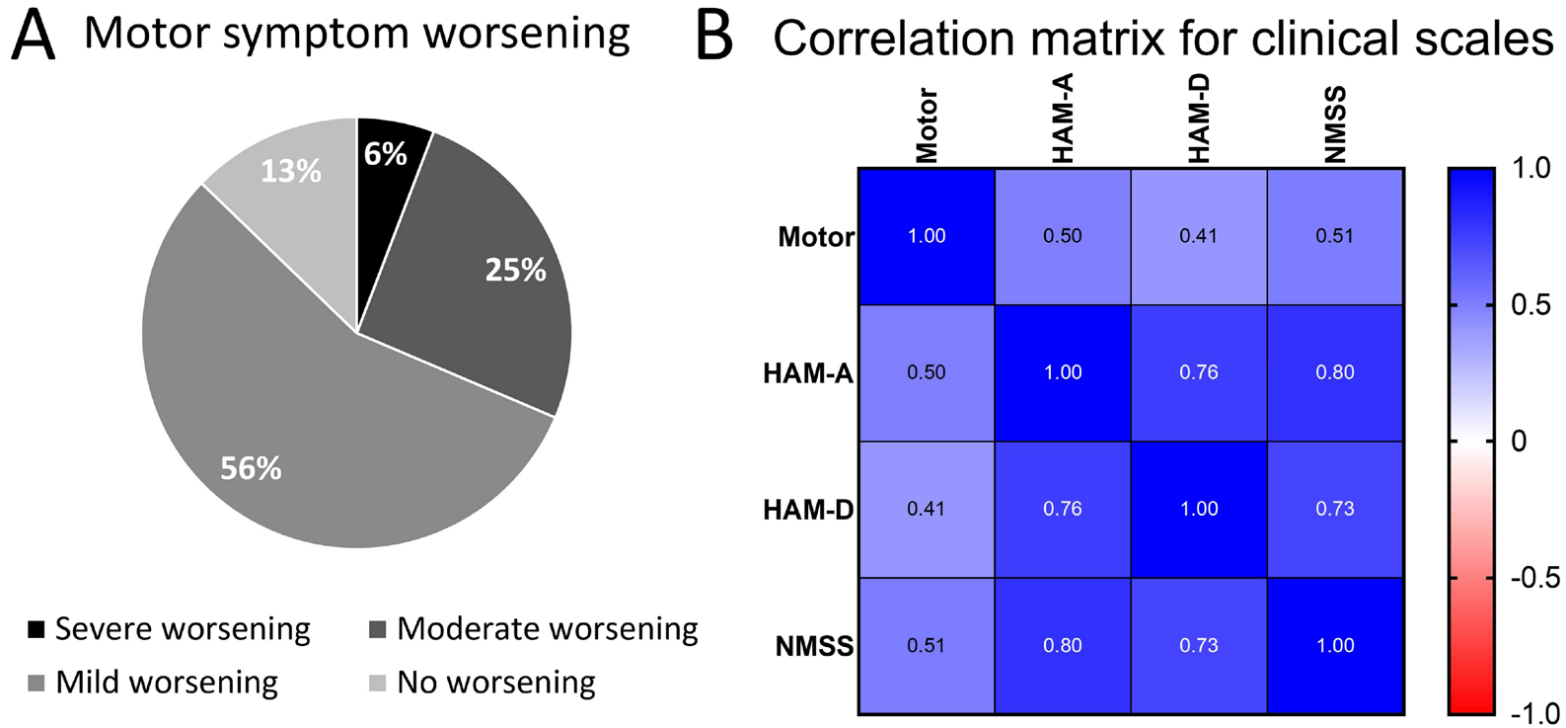
The prevalence of the levels of disease motor worsening (**A**) and correlation of worsening with clinical scales (**B**).

**Figure 3:**
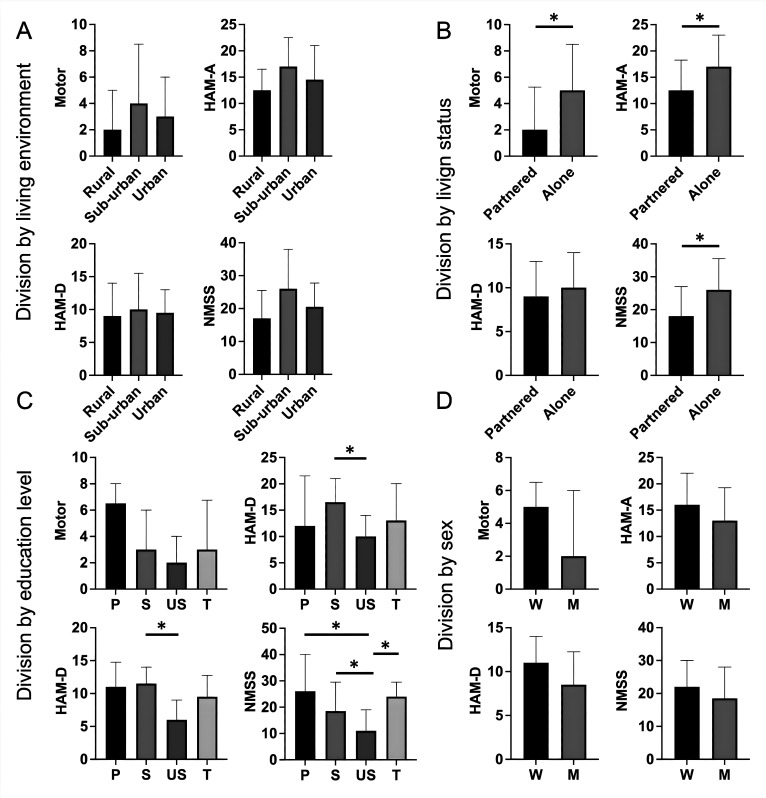
The association of clinical scale results and living environment (**A**); living status (**B**); educational level (**C**); and sex (**D**).

The majority of patients reported subjective worsening of motor symptoms (Figure 2). Motor symptoms worsening was not dependent on sex (U = 701.5; *P* = 0.053), age (U = 803; *P* = 0.1768), level of education (Z = 3.504; *P* = 0.320), settlement size (Z = 2.859; *P* = 0.239), duration of disease (Z = 3.039; *P* = 0.218), check-up avoidance (U = 439; *P* =0. 225), and Charlson comorbidity index categories (Z = 5.340; *P* = 0.069) (Table 2). However, patients who lived alone, as opposed to those living with a partner/family, had significant worsening of motor symptoms (*P* = 0.016) (Figure 3B).

**Table 2:** The associations between patients' characteristics and the presence of motor, anxiety, depression, and non-motor symptoms*

	Motor symptoms		HAM-A		HAM-D		NMSS	
	Median (IQR)	*P* Value	Median (IQR)	*P* Value	Median (IQR)	*P* Value	Median (IQR)	*P* Value
Sex								
male	2.00 (5.00)	0.053	13.00 (12.25)	0.676	8.50 (8.75)	0.338	18.50 (19.00)	0.249
female	5.00 (4.50)		16.00 (14.50)		11.00 (8.00)		22 (17.50)	
Age								
<60 years	4.00 (4.00)	0.177	15.00 (12.00)	0.239	9.00 (8.00)	0.468	19.00 (16.00)	0.358
>60 years	2.00 (5.00)		14.00 (14.50)		10.50 (9.75)		19.00 (21.75)	
Living status								
partnered/family	2.00 (4.25)	0.016	12.50 (11.25)	0.046	9.00 (8.00)	0.254	18.00 (18.25)	**0.043**
alone	5.00 (6.00)		17.00 (13.50)		14.00 (13.00)		26.00 (23.50)	
Level of education								
primary	6.50 (6.00)	0.320	12.00 (16.25)	0.045	11.00 (9.50)	0.200	26.00 (29.25)	**0.030**
secondary	3.00 (5.00)		16.50 (8.75)		11.50 (7.00)		18.50 (16.25)	
upper secondary	2.00 (3.00)		10.00 (10.00)		6.00 (6.00)		11.00 (14.00)	
tertiary	3.00 (4.75)		13.00 (13.75)		9.50 (8.75)		24.00 (21.00)	
Settlement size								
rural (0-10 000 inhabitants)	2.00 (4.00)	0.239	12.50 (9.75)	0.199	9.00 (9.25)	0.473	17.00 (15.75)	0.361
semiurban (10 000-100 000 inhabitants)	4.00 (5.50)		17.00 (15.00)		10.00 (10.00)		26.00 (27.00)	
urban (>100 000 inhabitants)	3.00 (5.00)		14.50 (14.00)		9.50 (8.00)		20.50 (19.50)	
Duration of disease (years)								
1 to 5	3.00 (5.00)	0.219	9.00 (11.00)	0.001	5.00 (9.00)	0.012	13.00 (15.00)	**0.048**
6 to 10	2.00 (5.00)		15.00 (16.00)		11.00 (11.00)		19.00 (22.00)	
11+	4.00 (5.00)		17.00 (11.00)		11.00 (8.00)		28.00 (21.50)	
Avoiding check-ups								
yes	3.50 (5.50)	0.225	18.50 (15.50)	0.119	10.50 (13.25)	0.280	23.00 (24.25)	**0.019**
no	3.00 (5.00)		14.00 (11.85)		9.00 (8.00)		18.50 (18.50)	
Charlson comorbidity index								
0 to 2	3.00 (4.00)	0.069	14.00 (11.00)	0.050	9.00 (8.00)	0.483	15.00 (15.00)	0.246
3 to 4	2.00 (5.00)		14.00 (14.00)		10.00 (10.00)		22.00 (22.00)	
5 to 7	5.00 (5.50)		17.00 (19.50)		11.00 (8.50)		27.00 (28.50)	

*Abbreviations: HAM-A – Hamilton Anxiety Rating Scale; HAM-D – Hamilton Depression Rating Scale; NMSS – Non-Motor Symptom Assessment Scale for Parkinson’s Disease, IQR – interquartile range.

**Figure 4:**
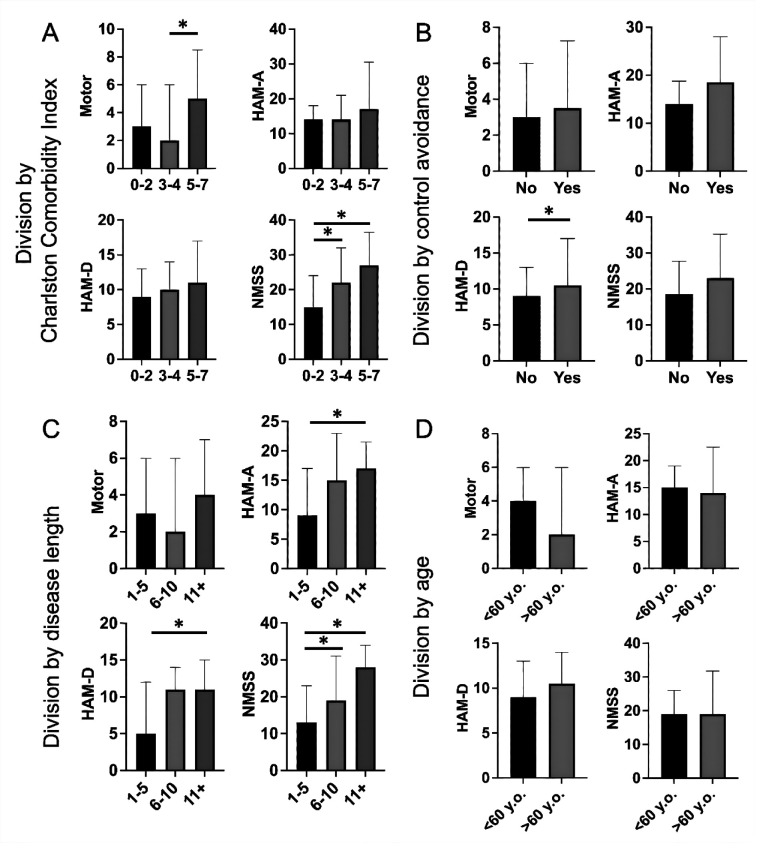
The associations of clinical scale results and Charlson comorbidity index (CCI) (**A**); avoidance of check-ups (**B**); disease length (**C**); and age (**D**).

The majority of our patients reported more frequent and severe anxiety symptoms (65.35%), while fewer patients reported problems with depression (45.88%). Non-motor symptoms were not significantly associated with sex (*P* = 0.676), age (U = 827.5; *P* = 0.239), settlement size (Z = 3.232; *P* = 0.198), and check-ups avoidance (U = 402.5; *P* = 0.119) (Table 2). However, non-motor symptoms significantly worsened in patients who lived alone (*P* = 0.046) (Figure 3B), had higher Charlson comorbidity index (Z = 3.097; *P* = 0.050) (Figure 4A), and had longer disease duration (Z = 3.831; *P* = 0.001) (Figure 4C). Patients with primary, secondary, and tertiary education had higher NMSS scores than those with upper secondary education (Z = 8.065; *P* = 0.045) (Figure 3C) (3).

Anxiety symptoms were not significantly associated with sex (*P* = 0.248), age (U = 867.5; *P* = 0.357), settlement size (Z = 1.031; *P* = 0.3612), and Charlson comorbidity index (Z = 1.426; *P* = 0.246). Anxiety symptoms significantly worsened in patients who lived alone (U = 592; *P* = 0.043) (Figure 3B), avoided check-ups (U = 327; *P* = 0.019) (Figure 4B), and had longer disease duration (Z = 6.076; *P* = 0.048) (Figure 4C). Patients with secondary education had significantly higher anxiety scores than those with upper secondary education (Z-value 2.906; *P* = 0.0037) (Figure 3C). Although the majority of our patients (n = 73) did not avoid check-ups, the patients who avoided them had significant worsening of anxiety symptoms (U = 327; *P* = 0.019) (Figure 4B). No significant findings were found in other categories.

Compared with motor and anxiety symptoms, patients less frequently reported depression symptom presence or worsening (Table 2). Depression symptoms were not significantly associated with sex (*P* = 0.338), age (*P* = 0.468), living status (*P* = 0.253), level of education (*P* = 0.199), settlement size (Z = 1.098; *P* = 0.682), check-ups avoidance (U = 453.5; *P* = 0.279), and Charlson comorbidity index (Z = 1.682; *P* = 0.483). Nevertheless, patients with longer duration of disease had significantly higher HAM-D scores (Z = 8.916; *P* = 0.012) (Figure 4C).

Motor worsening positively moderately correlated with non-motor worsening as measured by the HAM-A, HAM-D, and NMSS scales (motor/HAM-A r = 0.50; motor/HAM-D r = 0.41, motor/NMSS r = 0,51). There was a strong positive correlation between HAM-A, HAM-D and NMSS tests, which is not surprising as the NMSS part of the questionnaire includes overlapping questions on anxiety and depression (HAM-A/HAM-D r = 0.76; HAM-A/NMSS r = 0.80; HAM-D/NMSS r = 0.73 (Figure 2B).

## Discussion

This study had several important findings. First, most of our patients reported worsening of motor symptoms since the beginning of the pandemic. Patients who lived alone reported significantly greater worsening than those living with a partner. Second, the majority of our patients had anxiety problems. Higher HAM-A scores were observed in patients who lived alone, were less educated, had a longer disease duration, and avoided check-ups. Third, compared with motor and anxiety symptoms, fewer patients had problems with depression. Patients with longer duration of disease had significantly higher HAM-D scores. Fourth, many patients reported the worsening of non-motor symptoms. Higher NMSS scores were observed in patients who lived alone, were less educated, had a longer disease duration, and had higher Charlson comorbidity index.

The majority of our patients reported worsening of both motor and non-motor symptoms. The main cause for this could be a reduced opportunity to engage in physical activities due to the COVID-19 pandemic (8). Croatia had several lockdowns and implemented numerous restrictive measures, all of which drastically limited the everyday activities and the possibility to perform physical activity. Physical activity in PD patients is linked with improved quality of life and mood, better cognitive performance in several domains, and less motor and non-motor disturbances (13-15). Thus, less physical activity could be a risk factor for motor and non-motor symptoms worsening. The effect of the COVID-19 pandemic on exercise in PD patients has already been linked with subjective increase of motor and non-motor symptoms (15).

Non-motor symptoms are a significant burden in advanced Parkinson's disease, as they lead to significant disability, frequent hospital admissions, poor quality of life, and a shorter life duration (16). Home confinement during the pandemic was associated with new-onset or worsening of sleep disturbances in Parkinson's disease patients, which can deteriorate the disease symptoms and reduce the quality of life (17). Our results suggest that living alone, lower education, longer disease duration, and higher Charlson comorbidity index are risk factors for NMS worsening.

Patients who live alone, as opposed to patients living with a spouse or partner, showed a greater worsening of both motor and non-motor symptoms, such as anxiety. Social isolation due to the protective measures against the COVID-19 pandemic, made it even harder for these patients to maintain healthy social contacts. Fewer social contacts and living alone could eventually evoke the feeling of loneliness, which can be associated with a lower quality of life in Parkinson's disease patients (18). Loneliness, social isolation, and living alone increased the likelihood of death, after taking into consideration multiple covariates, by 26%, 29%, and 32%, respectively (19).

Anxiety and depression negatively affect the quality of life in patients with Parkinson's disease (20). These patients had a higher incidence of anxiety and depression during the pandemic (21). The higher incidence could be due to social isolation, fear of contracting COVID-19, or limited access to health care services. We found significantly higher anxiety scores in patients who lived alone, were less educated, had longer disease duration, and had avoided check-ups, while depression scores mostly depended on disease duration. The effect of disease duration on anxiety and depression could be explained by progressive deterioration of motor symptoms due to dopaminergic neuron loss. Motor symptoms in Parkinson's disease patients become more severe over the years, causing a higher degree of disability and requiring higher doses of medications, which positively correlates with anxiety and depression (22). Furthermore, because of impaired dopamine-dependent adaptation, patients with PD are susceptible to increased levels of psychological stress, which can worsen motor and non-motor symptoms of the disease, but also trigger psychiatric conditions such as depression and anxiety (8,9).

The psychological effect of the COVID-19 pandemic has resulted in more frequent avoidance of follow-up examinations. In the USA, 41% respondents avoided or delayed medical care due to COVID-19 concerns (9). Avoidance of follow-up examinations delays a timely recognition of symptoms worsening and an early treatment. Additionally, social restrictions interrupted routine treatment of neurological diseases. In a study by Zipprich et al (6), 31.3% of patients reported a loss in mobility due to restrictions, partly because of discontinuation of routine therapies. An Italian study (18) reported the greatest worsening in chronic neurological diseases requiring complex clinical treatment and frequent hospital controls, often by a multidisciplinary team (18).

Although the epidemiological situation in many countries calmed and vaccines have been introduced, many people still fear going to the hospital. This presents a great challenge in the treatment of not only Parkinson's disease patients, but of all chronic patients. Increased levels of cognitive stress, less physical activity, and poor disease control, due to examination avoidance or treatment interruption, create a fertile ground for worsening of Parkinson's disease symptoms and their negative effect on the quality of life.

The limitations of this study are the lack of a control group and the cross-sectional design. For this reason, we could not determine if social isolation affected Parkinson’s patients more than it affected the general population. Therefore, we only analyzed people with Parkinson’s based on certain characteristics that might be more affected by social isolation.

Based on our results, we can recommend the following: 1) patients who live alone during the pandemic and their families need to receive special care and consultation; 2) greater care should be given to patients who tend to avoid check-ups and they should be offered counseling due to increased anxiety prevalence; and 3) greater focus should be directed to non-motor symptoms as they positively correlate with motor symptoms.

The current pandemic has changed our society in ways that could pose a risk for patients with Parkinson’s disease. Further longitudinal studies are warranted to assess the effect of social isolation on the physical and mental health of people with Parkinson's disease and to identify the risk factors for disease exacerbation. These studies need to determine the areas where we should direct our care in Parkinson's disease patients during stressful times in order to achieve timely intervention and better disease control.
