# Supplementary material for: Effect of the coronavirus disease 2019 pandemic on people with Parkinson's disease: experience from a Croatian regional center
Source: Croat Med J. 2022 Feb;63(1):62–70. doi: 10.3325/cmj.2022.63.62 (PMC8895329; doi:10.3325/cmj.2022.63.62)
Supplement: Supplementary Figure 1 [file CroatMedJ_63_s007.pdf]

Parkinson's Disease Questionnaire - Telephone Survey

Sociodemographic questionnaire	
1. Age? (Expressed in years)	
2. Sex? (Male/Female)	
3. Disease duration? (Expressed in years)	
4. Level of education? (Primary education, Secondary education, Upper secondary education, Tertiary education)	
5. Relationship status (alone/partnered) / Living status (alone/partnered or family)?	
6. Size of habitat? (0 - 10,000 ,10 – 20,000, 20 – 50,000, 50 – 100,000, 100 – 500,000, more than 500,000 inhabitants)	
7. Employment status? (student, employed, unemployed, retired)	
8. Presence of comorbidities (diabetes, arterial hypertension, COPD etc.)?	
9. Have you been tested COVID-19 positive, since the start of the pandemic? (Yes/No)	
10. Did you avoid regular checkups with your doctor? (Yes/No)	

Questionnaire for motor symptoms	Self assessment 0-4
11. Did your tremor get worse?	
12. Is your rigor more pronounced than before?	
13. Do you have more trouble walking?	
14. Did you have more problems with falls?	

0 - symptom did not worsen, 1 - mild worsening, 2 - moderate worsening, 3 - severe worsening, 4 - extremely severe worsening

Hamilton Anxiety Rating Scale

0 - not present, 1 - slightly present, 2 - moderately present, 3 - very present, 4 - extremely present

Question	0 - 4
1. Anxious mood: Worries, anticipation of the worst, fearful anticipation, irritability.	
2. Tension: Feelings of tension, fatigability, startle response, moved to tears easily, trembling, feelings of restlessness, inability to relax.	
3. Fears: Of dark, of strangers, of being left alone, of animals, of traffic, of crowds.	
4. Insomnia: Difficulty in falling asleep, broken sleep, unsatisfying sleep and fatigue on waking, dreams, nightmares, night terrors.	
5. Intellectual: Difficulty in concentration, poor memory.	
6. Depressed mood: Loss of interest, lack of pleasure in hobbies, depression, early waking, diurnal swing.	
7. Somatic (muscular): Pains and aches, twitching, stiffness, myoclonic jerks, grinding of teeth, unsteady voice, increased muscular tone.	
8. Somatic (sensory): Tinnitus, blurring of vision, hot and cold flushes, feelings of weakness, pricking sensation.	
9. Cardiovascular symptoms: Tachycardia, palpitations, chest pain, throbbing of vessels, fainting feelings, missing beat.	
10. Respiratory symptoms: Pressure or constriction in chest, choking feelings, sighing, dyspnea.	
11. Gastrointestinal symptoms: Difficulty in swallowing, wind abdominal pain, burning sensations, abdominal fullness, nausea, vomiting, borborygmi, looseness of bowels, weight loss, constipation.	
12. Genitourinary symptoms: Frequency of micturition, urgency of micturition, amenorrhea, menorrhagia, development of frigidity, premature ejaculation, loss of libido, impotence.	
13. Autonomic symptoms: Dry mouth, flushing, pallor, tendency to sweat, giddiness, tension headache, raising of hair.	
14. Behavior at interview: Fidgeting, restlessness or pacing, tremor of hands, furrowed brow, strained face, sighing or rapid respiration, facial pallor, swallowing, etc.	

Hamilton Depression Rating Scale

1. DEPRESSED MOOD (sadness, hopeless, helpless, worthless)
   0 | __ | Absent.
   1 | __ | These feeling states indicated only on questioning.
   2 | __ | These feeling states spontaneously reported verbally.
   3 | __ | Communicates feeling states non-verbally, i.e. through facial expression, posture, voice and tendency to weep.
   4 | __ | Patient reports virtually only these feeling states in his / her spontaneous verbal and non-verbal communication

2. FEELINGS OF GUILT
   0 | __ | Absent.
   1 | __ | Self reproach, feels he / she has let people down.
   2 | __ | Ideas of guilt or rumination over past errors or sinful deeds.
   3 | __ | Present illness is a punishment. Delusions of guilt.
   4 | __ | Hears accusatory or denunciatory voices and / or experiences threatening visual hallucinations.

3. SUICIDE
   0 | __ | Absent.
   1 | __ | Feels life is not worth living.
   2 | __ | Wishes he / she were dead or any thoughts of possible death to self.
   3 | __ | Ideas or gestures of suicide.
   4 | __ | Attempts at suicide (any serious attempt rate 4).

4. INSOMNIA: EARLY IN THE NIGHT
   0 | __ | No difficulty falling asleep.
   1 | __ | Complains of occasional difficulty falling asleep, i.e. more than 1⁄2 hour.
   2 | __ | Complains of nightly difficulty falling asleep

5. INSOMNIA: MIDDLE OF THE NIGHT
   0 | __ | No difficulty.
   1 | __ | Patient complains of being restless and disturbed during the night.
   2 | __ | Waking during the night - any getting out of bed rates
   2 (except for purposes of voiding)

6. INSOMNIA: EARLY HOURS OF THE MORNING
   0 |__| No difficulty.
   1 |__| Waking in early hours of the morning but goes back to sleep.
   2 |__| Unable to fall asleep again if he/she gets out of bed.

7. WORK AND ACTIVITIES
   0 |__| No difficulty.
   1 |__| Thoughts and feelings of incapacity, fatigue or weakness related to activities, work or hobbies.
   2 |__| Loss of interest in activity, hobbies or work – either directly reported by the patient or indirect in listlessness, indecision and vacillation (feels he/she has to push self to work or activities).

3 |__| Decrease in actual time spent in activities or decrease in productivity. Rate 3 if the patient does not spend at least three hours a day in activities (job or hobbies) excluding routine chores.
4 |__| Stopped working because of present illness. Rate 4 if patient engages in no activities except routine chores, or if patient fails to perform routine chores unassisted.

8. RETARDATION (slowness of thought and speech, impaired ability to concentrate, decreased motor activity)
0 |__| Normal speech and thought.
1 |__| Slight retardation during the interview.
2 |__| Obvious retardation during the interview.
3 |__| Interview difficult.
4 |__| Complete stupor.

9. AGITATION
0 |__| None.
1 |__| Fidgetiness.
2 |__| Playing with hands, hair, etc.
3 |__| Moving about, can’t sit still.
4 |__| Hand wringing, nail biting, hair-pulling, biting of lips.

10. ANXIETY PSYCHIC
0 |__| No difficulty.
1 |__| Subjective tension and irritability.
2 |__| Worrying about minor matters.
3 |__| Apprehensive attitude apparent in face or speech.
4 |__| Fears expressed without questioning.

11. ANXIETY SOMATIC (physiological concomitants of anxiety) such as: gastro-intestinal– dry mouth, wind, indigestion, diarrhea, cramps, belching cardio-vascular – palpitations, headaches respiratory – hyperventilation, sighing urinary frequency sweating
0 |__| Absent.
1 |__| Mild.
2 |__| Moderate.
3 |__| Severe.
4 |__| Incapacitating.

12. SOMATIC SYMPTOMS GASTRO-INTESTINAL
0 |__| None.
1 |__| Loss of appetite but eating without staff encouragement. Heavy feelings in abdomen.
2 |__| Difficulty eating without staff urging. Requests or requires laxatives or medication for bowels or medication for gastro-intestinal symptoms.

13. GENERAL SOMATIC SYMPTOMS
0 |__| None.
1 |__| Heaviness in limbs, back or head. Backaches, headaches, muscle aches. Loss of energy and

fatigability.
2 |__| Any clear-cut symptom rates 2.

14. GENITAL SYMPTOMS (symptoms such as loss of libido, menstrual disturbances)
0 |__| Absent.
1 |__| Mild.
2 |__| Severe.

15. HYPOCHONDRIASIS
0 |__| Not present.
1 |__| Self-absorption (bodily).
2 |__| Preoccupation with health.
3 |__| Frequent complaints, requests for help, etc.
4 |__| Hypochondriacal delusions.

16. LOSS OF WEIGHT (RATE EITHER a OR b)

a) According to the patient	b) According to weekly measurements:
0 |__| No weight loss.	0 |__| Less than 1 lb weight loss in week.
1 |__| Probable weight loss associated with present illness.	1 |__| Greater than 1 lb weight loss in week.
2 |__| Definite (according to patient) weight loss.	2 |__| Greater than 2 lb weight loss in week
3 |__| Not assessed.	3 |__| Not assessed.

17. INSIGHT
0 |__| Acknowledges being depressed and ill.
1 |__| Acknowledges illness but attributes cause to bad food,climate, overwork, virus, need for rest, etc.
2 |__| Denies being ill at all.

Non-motor symptom questionnaire (Adapted from Chaudhuri KR)

Question	Yes/No	Worsening 1 - 4
**Domain 1: Cardiovascular including falls**		
1. Does the patient experience light-headedness, dizziness, weakness on standing from sitting or lying position?		
2. Does the patient fall because of fainting or blacking out?		
**Domain 2: Sleep / fatigue**		
3. Does the patient doze off or fall asleep unintentionally during daytime activities? (For example, during conversation, during mealtimes, or while watching television or reading).		
4. Does fatigue (tiredness) or lack of energy (not slowness) limit the patient’s daytime activities?		
5. Does the patient have difficulties falling or staying asleep?		
6. Does the patient experience an urge to move the legs or restlessness in legs that improves with movement when he/she is sitting or lying down inactive?		
**Domain 3: Mood / cognition**		
7. Has the patient lost interest in his/her surroundings?		
8. Has the patient lost interest in doing things or lack motivation to start new activities?		
9. Does the patient feel nervous, worried or frightened for no apparent reason?		
10. Does the patient seem sad or depressed or has he/she reported such feelings?		
11. Does the patient have flat moods without the normal “highs” and “ lows”?		
12. Does the patient have difficulty in experiencing pleasure from their usual activities or report that they lack pleasure?		
**Domain 4: Perceptual problems/hallucinations**		
13. Does the patient indicate that he/she sees things that are not there?		

14. Does the patient have beliefs that you know are not true? (For example, about being harmed, being robbed or being unfaithful)		
15. Does the patient experience double vision? (2 separate real objects and not blurred vision)		
**Domain 5: Attention/memory**		
16. Does the patient have problems sustaining concentration during activities? (For example, reading or having a conversation)		
17. Does the patient forget things that he/she has been told a short time ago or events that happened in the last few days?		
18. Does the patient forget to do things? (For example, take tablets or turn off domestic appliances?)		
**Domain 6: Gastrointestinal tract**		
19. Does the patient dribble saliva during the day?		
20. Does the patient have difficulty swallowing?		
21. Does the patient suffer from constipation? (Bowel action less than three times weekly)		
**Domain 7: Urinary**		
22. Does the patient have difficulty holding urine? (Urgency)		
23. Does the patient have to void within 2 hours of last voiding? (Frequency)		
24. Does the patient have to get up regularly at night to pass urine? (Nocturia)		
**Domain 8: Sexual function**		
25. Does the patient have altered interest in sex?<br>(Very much increased or decreased, please underline)		
26. Does the patient have problems having sex?		
**Domain 9: Miscellaneous**		
27. Does the patient suffer from pain not explained by other known conditions? (Is it related to intake of drugs and is it relieved by antiparkinson drugs?)		
28. Does the patient report a change in ability to taste or smell?		

29. Does the patient report a recent change in weight (not related to dieting)?		
30. Does the patient experience excessive sweating (not related to hot weather)?		
